# Advanced Care Planning of Unmarried and Childless Older U.S. Adults: Are They Less Prepared for the End-of-Life?

**DOI:** 10.1093/geroni/igaf122.147

**Published:** 2025-12-31

**Authors:** Deborah Carr, Shinae Choi

**Affiliations:** Boston University, Boston, Massachusetts, United States; The University of Alabama, Tuscaloosa, Alabama, United States

## Abstract

Planning for end-of-life health care is critical for dying persons and their families they leave behind. Advance care planning (ACP), which encompasses an advance directive and informal discussions, is an important step toward the receipt of end-of-life medical care that aligns with one’s wishes. ACP is associated with better psychological adjustment and family functioning among the decedent’s survivors. However, it is unclear how those growing old outside of traditional marriage or who have complex childbearing histories prepare for end-of-life. We use data from the 2016-2022 waves of the Health and Retirement Study (N = 5,295) to explore how complex romantic partnership histories and parental status affect four subtypes of ACP (advance directives only [formal], informal discussions only [informal], both, or neither). We examine the extent to which these patterns differ by gender, after adjusting for socioeconomic, health, and psychosocial factors that have been found elsewhere to affect ACP. Multivariable analyses show that divorced and widowed persons, and especially recently widowed persons, are most likely to do formal ACP and both formal/informal ACP, with the magnitude of these patterns differing slightly by gender. Never-married persons are least likely to do ACP. Childless persons have especially low rates of ACP. Parents are more likely than childless persons to have informal end-of-life discussions only. Our results reveal the complex ways that family statuses affect end-of-life preparations and can inform policies and professional strategies to encourage these practices.

